# Iatrogenic fibrotic encapsulation of NANCE palatal arch appliance in the hard palate and its surgical management: a case report

**DOI:** 10.1093/jscr/rjad120

**Published:** 2023-03-27

**Authors:** Abimbola Y Adeloye, Uchenna P Egbunah, Ifeoma L Utomi

**Affiliations:** Department of Orthodontics, Child Dental Health, Lagos University Teaching Hospital, Lagos, Nigeria; Department of Oral and Maxillofacial Surgery, Lagos University Teaching Hospital, Lagos, Nigeria; Department of Orthodontics, Child Dental Health, College of Medicine, University of Lagos/Lagos University Teaching Hospital, Lagos, Nigeria

## Abstract

Fibrosis is the overgrowth of connective tissue resulting from chronic inflammatory reactions induced by persistent tissue injury such as iatrogenic injury from prolonged use of orthodontic appliances. We report a case of a 19-year-old female who presented with a complaint of dental malocclusion. Her first presentation was 5 years prior when she received a Nance palatal arch appliance. However, she did not keep to her follow-up appointments and could not complete her treatment. Intraoral examination revealed the Nance palatal arch appliance completely buried in fibrotic tissue of the hard palate. The appliance was resistant to removal by conventional means and surgical exposure and removal were performed. A new Nance palatal arch appliance was fabricated and fitted and the patient continued to receive further orthodontic treatment. This report elucidates the importance of regular dental appointments for patients on orthodontic therapy to prevent complications and minimize the need for surgical interventions.

## INTRODUCTION

Fibrosis is defined as the overgrowth, hardening and/or scarring of connective tissue and it represents the end result of chronic inflammatory reactions [1]. Iatrogenic injury refers to tissue damage from necessary medical, surgical or pharmacological interventions [2]. Orthodontic treatment is one of the most common causes of iatrogenic injury to the tongue, gingivae and oral mucosa [3]. The application of constant pressure over the orthodontic appliance traumatizes adjacent oral soft tissues. In addition, orthodontic appliances serve as plaque retentive factors promoting plaque and bacterial action on the gingivae and oral mucosa. The continuous injury and plaque action on oral soft tissues result in chronic inflammation which may progress to fibrosis of the oral mucosa and in rare cases, the offending appliance can get embedded into the oral mucosal and gingival tissues [4]. In this article, we report a rare case of iatrogenic trauma with fibrotic encapsulation of a Nance palatal arch appliance in the palatal mucosa and the surgical management.

## CASE REPORT

**Figure 1:**
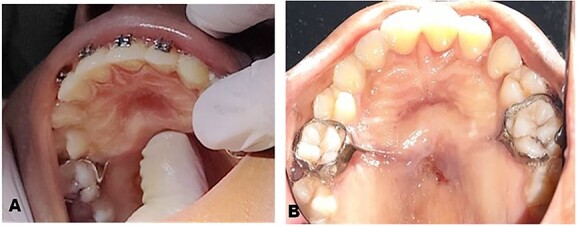
Preoperative clinical photographs: (**A**) Preoperative clinical picture showing clinical examination of the palate. (**B**) Preoperative clinical picture showing buried Nance palatal arch appliance after administration of LA.

A 19-year-old female presented at the Orthodontic clinic of the Lagos University Teaching Hospital, Lagos, Nigeria with a complaint of dental malocclusion. Her first presentation to the orthodontics clinic, Lagos University Teaching Hospital was 5 years prior when she received orthodontic treatment in the form of upper and lower preadjusted edgewise fixed orthodontic appliance and Nance palatal arch appliance. However, patient did not keep to her follow-up orthodontic appointments for availability reasons and presented back to our orthodontic clinic 5 years after initial treatment for continuation of treatment. On intraoral examination, the hard palate appeared thick and fibrotic but with an overall clinically healthy appearance. The bands of the Nance palatal arch appliance were fixed satisfactorily to the upper first molars but the looped 0.9 mm hard stainless steel wire and the acrylic button of the Nance palatal arch appliance were seen to be completely buried in fibrotic palatal tissue of the hard palate (Fig. 1A).

The clinical history and examination suggested fibrotic encapsulation of the Nance palatal arch appliance secondary to prolonged use. The appliance was resistant to removal by conventional means since it was embedded firmly in the palatal mucosa and subsequently surgical exposure and removal of Nance palatal arch appliance were planned (Fig. 1B). Before surgical removal, the patient was pre-medicated with oral antibiotics and analgesics to prevent super-infection and aid pain management. Medications used included 500 mg amoxicillin, 400 mg metronidazole and 400 mg ibuprofen 30 min before surgical procedure.

**Figure 2:**
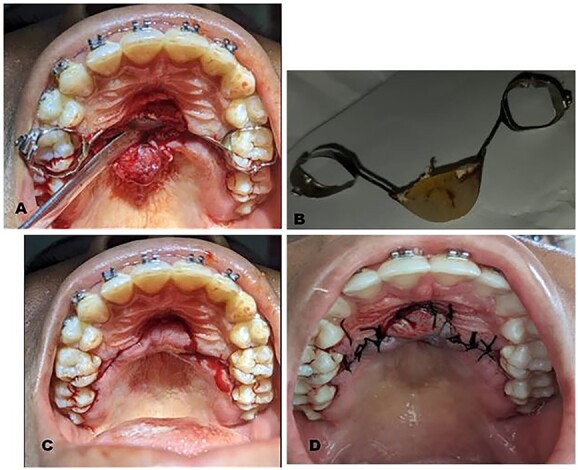
Intraoperative clinical photographs: (**A**) Trans-surgical view: partial thickness palatal mucosa flap raised using a periosteal elevator to expose buried Nance appliance. (**B**) Nance appliance delivered out of the palatal mucosa. (**C**) Trans-surgical view: raised flap repositioned and showing the outline of incision made. (**D**) Repositioned flap sutured using black silk sutures.

**Figure 3:**
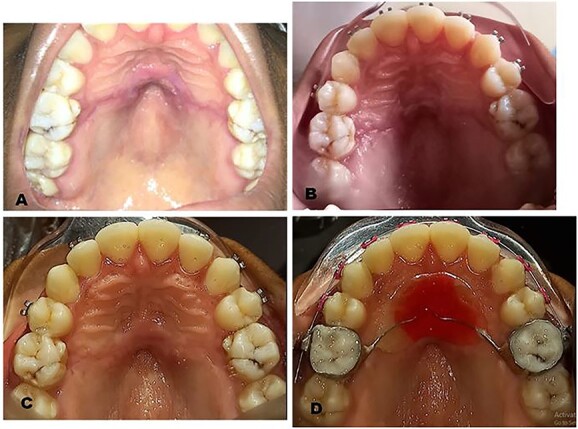
Postoperative clinical photographs: (**A**) One week postoperative photograph after suture removal. (**B**) Two weeks postoperative photograph. (**C**) Four weeks postoperative photograph. (**D**) Six weeks postoperative photograph after fitting of new Nance appliance.

The surgical operation was performed using local anesthesia (LA). Two LA cartridges containing 2% lidocaine were administered on the palate using the subperiosteal infiltration technique. Incision was made on the palatal mucosa following the curvature of the Nance appliance using a No. 15 surgical blade. A partial thickness palatal mucosae flap was raised using a periosteal elevator to expose the buried stainless steel wire and clear acrylic palatal button of the Nance appliance (Fig. 2A). Molar bands on teeth 16 and 26 were removed using a band remover and the periosteal elevator was used to deliver the Nance appliance out of the palatal mucosa (Fig. 2B). The raised flap was repositioned (Fig. 2C) and sutured using black silk suture (Fig. 2D) and hemostasis was achieved by pressure application (patient was asked to bite on gauze for 5 min). The patient was placed on 500 mg amoxicillin, 400 mg metronidazole and 400 mg ibuprofen three times daily for 5 days postoperatively. Postoperative warm saline mouth rinse eight times daily for 2 weeks was also recommended. The patient was followed up 24 h postoperatively (PO), 7 days PO (Fig. 3A), 2 weeks PO (Fig. 3B) and 4 weeks PO (Fig. 3C). Sutures were removed 7 days PO. The patient’s postoperative course was uneventful. A new Nance palatal arch appliance with 0.9 mm hard stainless steel wire loops and the tinted acrylic button was fabricated and patient was fitted with the new Nance appliance 6 weeks PO (Fig. 3D). The patient continued to be on regular follow-up and received further dental and orthodontic treatment.

## DISCUSSION

One of the major causes of iatrogenic physical trauma to oral tissues during orthodontic treatment is irritation from ill-fabricated appliances [4]. Traumatic injuries of physical means can occur on the palate due to ill-fitted orthodontic appliance used to reinforce anchorage [4]. In the present case, the Nance appliance was not ill-fabricated or ill-fitted; however, it was used for a prolonged time due to inability of the patient to meet up with her review appointments. The constant upward pressure from the tongue on a palatal appliance during mastication and speech over a long period of time plus fibrotic hyperplasia of the palatal mucosa secondary to chronic irritation has been shown to result in impaction of orthodontic appliances in the palatal mucosa [5] and may explain the resulting outcome of the appliance in this case.

Orthodontic appliances can inflict such injuries when fitted over a long period of time without review [3]. Orthodontists can increase their patient’s oral hygiene awareness by educating their patients on the importance of maintaining good oral hygiene practices. This would not only reduce the prevalence and severity of iatrogenic damage but would increase the long-term benefits of the orthodontic therapy [5]. The extent of involvement of the periodontal tissues will determine the preferred treatment option in cases of fibrosis [6]. Treatment could range from conventional removal of the causal agent to surgical intervention as seen in the case described. Orthodontic treatment has numerous benefits to patients in terms of improved aesthetics, function, oral health and self-esteem but it can also cause undesirable complications if patients do not adhere to their regular reviews and adequate oral healthcare is not implemented [7]. The orthodontist must ensure that the patient is aware of the possible complications and emphasize the role of the patient in the prevention of these undesirable outcomes [7].

## CONCLUSION

Prevention of complications in orthodontics is the responsibility of both the orthodontist and the patient and can be achieved if recommended regular dental appointments are observed. Routine dental checkups are important to prevent complications of orthodontic treatment thereby, minimizing the need for surgical interventions.

## Data Availability

The datasets used during the current study can be made available from the corresponding author on reasonable request.
